# Impairment of Hypoxia-Induced CA IX by Beta-Blocker Propranolol—Impact on Progression and Metastatic Potential of Colorectal Cancer Cells

**DOI:** 10.3390/ijms21228760

**Published:** 2020-11-19

**Authors:** Monika Barathova, Katarina Grossmannova, Petra Belvoncikova, Veronika Kubasova, Veronika Simko, Rudolf Skubla, Lucia Csaderova, Jaromir Pastorek

**Affiliations:** 1 Biomedical Research Centre, Department of Tumor Biology, Institute of Virology, Slovak Academy of Sciences Dubravska cesta 9, 84505 Bratislava, Slovakia; monika.barathova@savba.sk (M.B.); katarina.laposova@savba.sk (K.G.); petra.belvoncikova@savba.sk (P.B.); veronika.kubasova@savba.sk (V.K.); virunica@savba.sk (V.S.); jaromir.pastorek@gmail.com (J.P.); 2 Ruzinov Hospital, University Hospital Bratislava, Ruzinovska 6, 82101 Bratislava, Slovakia; skubla@ru.unb.sk; 3 Faculty of Medicine, Slovak Medical University, Limbová 12, 83303 Bratislava, Slovakia

**Keywords:** carbonic anhydrase IX, propranolol, beta-adrenoreceptors, spheroids, mitochondria

## Abstract

The coexistence of cancer and other concomitant diseases is very frequent and has substantial implications for treatment decisions and outcomes. Beta-blockers, agents that block the beta-adrenergic receptors, have been related also to cancers. In the model of multicellular spheroids formed by colorectal cancer cells we described a crosstalk between beta-blockade by propranolol and tumour microenvironment. Non-selective beta-blocker propranolol decreased ability of tumour cells to adapt to hypoxia by reducing levels of HIF1α and carbonic anhydrase IX in 3D spheroids. We indicated a double action of propranolol in the tumour microenvironment by inhibiting the stability of HIF1α, thus mediating decrease of CA IX expression and, at the same time, by its possible effect on CA IX activity by decreasing the activity of protein kinase A (PKA). Moreover, the inhibition of β-adrenoreceptors by propranolol enhanced apoptosis, decreased number of mitochondria and lowered the amount of proteins involved in oxidative phosphorylation (V-ATP5A, IV-COX2, III-UQCRC2, II-SDHB, I-NDUFB8). Propranolol reduced metastatic potential, viability and proliferation of colorectal cancer cells cultivated in multicellular spheroids. To choose the right treatment strategy, it is extremely important to know how the treatment of concomitant diseases affects the superior microenvironment that is directly related to the efficiency of anti-cancer therapy

## 1. Introduction

The presence of co-existing diseases and their often chronic treatment can significantly affect the proliferation, growth and metastasis of the tumour itself. Concomitant diseases may have a direct impact on cancer care, treatment selection and therapy effectiveness [1,2,3]. However, the evidence of the effect of comorbidities and their treatment on the outcome of anticancer therapy is missing, partly due to the lack of clinical studies from which such patients are often excluded [4]. As number of patients diagnosed with cancer increases and the population ages, management of comorbidities should play an important role. Cancer patients represent a population predisposed to the development of many comorbidities that affect the overall outcome [5]. Anticancer therapy alone is often associated with the development of cardiovascular diseases, hypertension, cardiotoxicity [6]. Epidemiological studies have shown that in the US four out of ten patients diagnosed with cancer (older than 65 years) suffer from at least one other chronic disease and 15% have two or more comorbidities [7]. However, the evidence of the effect of comorbidities and their treatment on the outcome of anticancer treatment is missing, also due to the lack of clinical studies from which such patients are often excluded [4]. The chronic treatment of comorbidities and the burden of comorbid diseases in relation to treatment and outcomes of cancer have been seldomly described.

**Figure 1:**
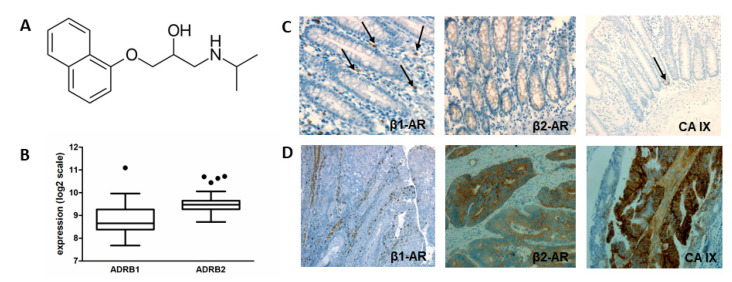
Evaluation of β1 and β2-AR expression and immunohistochemical analysis of CA IX and β AR expression in healthy colon (**C**) and colorectal cancer tissue samples (**D**). (**A**) Chemical structure of propranolol. (**B**) Box plot graph of the results of in silico analysis of β1 and β2-AR expression in colorectal neoplasms. Using the Genevestigator database we showed expression β1 and β2-AR at medium level in tissues derived from colorectal carcinoma. (**C**) The expression of β1 AR is associated with mastocytes (indicated by arrows) and smooth muscle of vessels in healthy colon tissues, expression of β2 AR was detected in cryptal cells. Weak expression of CA IX was found in cryptal cells (indicated by arrow). (**D**) The expression of β1 AR in human colorectal cancer samples was associated with mastocytes and smooth muscle of vessels in non-malignant regions of colorectal cancer (CRC), expression of β2 AR was detected in malignant cryptal cells. The strong expression of CA IX was localized to the cryptal cells.

Beta-blockers, agents that block the adrenergic β-receptors, have been used for decades in the treatment of cardiovascular disorders (CVD). They represent a specific class of drugs with a competitive antagonist effect on β-adrenoreceptors (β-AR). In the ESMO guidelines, selected beta-blockers are recommended as an agent to reduce the risk of cardiotoxicity [8]. Propranolol (Figure 1A) is one of the oldest known nonselective beta-blockers. This drug is used alone or together with other drugs to treat high blood pressure (hypertension), severe chest pain (angina), migraine headaches. Propranolol (PROP) has also been licensed by the EMA and FDA for the treatment of infantile haemangioma. The clinical trial data supporting the off-label use of PROP in different conditions, including haemorrhage, sepsis and hypermetabolic syndrome [9], akathisia associated with Alzheimer’s disease or psychosis [10] and aggression associated with brain injury or disease [11].

Several studies have confirmed that the use of beta-blockers can positively influence the treatment of tumours [12,13,14,15] and that β-AR signalling is involved in tumorigenesis and metastasis [16]. Epidemiologic studies revealed consistent relationships between stressful conditions and progression of already-incident tumours [17,18]. Retrospective studies revealed an association of β-blocker treatment with increased overall and progression-free survival in patients with prostate [19,20,21], breast [22,23,24,25], ovarian cancer [14] or melanoma [12,26]. A meta-analysis of 20,898 cancer patients indicated that usage of β-blocker may associate with prolonged survival in early stage cancer patients undergoing surgical resection [27]. However, these observations are in contrast to other studies involving also patients with different tumours–breast [28], melanoma [29,30] and renal cell carcinoma [31], where beta-blocker use was not associated with reduced risk of death or decreased cancer progression. A meta-analysis of epidemiological and perioperative clinical studies [32] found that beta-blocker use had no effect on cancer recurrence, disease-free survival or overall survival in patients with cancer. These contradictory results may stem from the heterogeneity of the patient groups, tumour types, stages and therapies as well as from differences in the type and duration of beta-blocker usage.

The downstream adrenergic signalling responses activated by stress are dependent on the expression of specific β-AR in various organs and tissues participating in the alert reaction [33]. Catecholamines, that is, adrenaline and noradrenaline, act via adrenergic receptors of α and β types (each with several subtypes), which are located in the plasma membrane and interact with sub-membrane heterotrimeric G proteins. Type β-adrenoreceptors can activate one of two G proteins Gs and Gi, which differentially regulate adenylate cyclase (Gs↑ a Gi↓), an enzyme converting ATP to cAMP, which then activates protein kinase A (PKA). PKA can phosphorylate and regulate a whole spectrum of proteins. In response to stress in tumours, catecholamines adrenaline and noradrenaline are distributed into tumour microenvironment [34] via circulating blood and norepinephrine is released from local sympathetic nerve fibres.

The microenvironment of a developing tumour is very heterogeneous and responds also to signals from the surrounding non-tumour environment. One of the important proteins influencing the tumour microenvironment is carbonic anhydrase IX (CA IX). CA IX belongs to the family of α carbonic anhydrases that catalyse a reversible hydration of carbon dioxide to bicarbonate ions and protons [35]. CA IX is a tumour-associated, hypoxia-regulated cell surface glycoprotein with pro-survival and pro-metastatic competence, activated by microenvironmental stresses. This membrane protein is involved in the adaptation to acidosis and supports tumour progression *via* its catalytic activity and/or non-catalytic functions [36]. As a part of the transport metabolone CA IX contributes to the maintenance of intracellular pH homeostasis and by its enzymatic activity it significantly contributes to the acidification of the tumour extracellular environment. This protein is regulated by hypoxia at several levels. Transcriptional activation of CA IX depends on hypoxia, especially on HIF1 that binds to the promoter immediately upstream of the transcription start and HIF1 is thus the primary transcriptional regulator of the gene [37]. This is reflected in the typical regional expression pattern of CA IX throughout a relatively broad perinecrotic zone in most of the hypoxic tumours and in the diffuse expression pattern in renal carcinomas where hypoxia inducible factor (HIF) is constitutively active due to a genetic inactivation of its negative regulator pVHL. In addition, hypoxia activates CA IX at the functional level through cAMP and the activation of protein kinase A, which phosphorylates Thr443 in the intracellular domain. Phosphorylation of Thr443 then mediates inside-out signalling to the extracellular catalytic domain that leads to the activation of the CA IX catalytic performance [38]. Furthermore, EGF-induced phosphorylation of Tyr449 is involved in a downstream activation of PI3K/Akt pathway [39]. CA IX can internalize from the cell surface to the cytosol via endocytosis. This phenomenon is activated by physiological stresses including hypoxia and calcium depletion as well as by specific antibodies binding to its extracellular domain [40,41]. The extracellular part of CA IX can be cleaved by metalloproteinase ADAM17 and released to the microenvironment in response to hypoxia, acidosis, chemotherapeutic drugs or inhibitors of carbonic anhydrases [42,43]. CA IX is an excellent therapeutic target because it belongs to very few cell surface proteins predominantly expressed in tumours and almost absent in healthy tissues. Its expression in non-cancerous tissues is rare and generally localized to epithelia of the stomach, gallbladder, pancreas and intestine [44]. This protein is an essential component of cells adaptation to hypoxia and acidosis and it displays unique characteristics that predestine its use in clinical practice during diagnostics as a biomarker, prognostic indicator and predictive factor for a broad range of solid tumours but also in anti-cancer therapy [36].

On the other hand, the mechanisms of the influence of beta-blockers on the tumour microenvironment have not yet been fully elucidated. One of the best known non-selective beta-blockers is propranolol, which has long been used in the treatment of heart failure. Propranolol not only affects cardiomyocytes, regulates blood pressure but also affects several parameters of tumour cells. The effects of propranolol on proliferation, apoptosis, migration, angiogenesis of tumour cells, and immune-related mechanisms were summarized and described in Pantziarka et al. (2016) [45]. Already in 2012, Chim et al. described suppressive effects of propranolol on haemangiomas through the HIF1α-VEGF-A angiogenesis axis and impact of propranolol on the PI3/Akt and p38/MAPK pathways [46]. The hypothesis that β-ARs are part of the HIF-regulating pathway for hypoxia sensing was tested by Cheong et al. (2016) [47]. Recent studies have shown that propranolol blocks autophagy and induces apoptosis when administered together with inhibitors of glycolysis, it inhibits mitochondrial bioenergetics, attenuates tumour metabolism and reduces tumour cell proliferation [48,49,50].

In our work, we describe a double action of propranolol in the tumour microenvironment by inhibiting the stability of HIF1α and thus mediating decrease of CA IX expression and, at the same time, by its effect on CA IX activity by decreasing the activity of PKA and consequences on proliferation and migration of tumour cell in 3D model of spheroids. We also demonstrate the effect of propranolol on the number of mitochondria in colorectal tumour cells and its impact on the level of oxidative phosphorylation enzymes.

## 2. Results

### 2.1. Expression of Beta Adrenoreceptors in Colorectal Carcinomas-In Silico Analysis

To investigate grounds behind the effect of propranolol on colorectal tumour cells, firstly we performed in silico analysis of β-adrenoreceptors expression in colorectal neoplasms using Genevestigator. Since this database integrates a spectrum of various microarrays and RNA-sequencing experiments, it allowed us to compare gene expressions across different biological contexts. The results of patients’ samples analyses showed that tissues derived from colorectal carcinoma (CRC) express β1 and β2-AR at medium levels (Figure 1B).

### 2.2. Expression of β-AR in Colorectal Carcinoma Tissues

Comorbidities treated with beta-blockers often occur in patients with CRC. Therefore, we focused on the investigation of β-AR expression in tumour tissue of CRC patients. In paraffin sections of CRC tumours, we immunohistochemically analysed CA IX expression as well as the expression of β1 and β2 adrenoreceptors. We were interested not only in their expression but also in their localization within tissue. We found association of the β1-AR expression with mastocytes and smooth muscle of vessels in non-malignant regions of tissues (Figure 1D). The expression of β2-AR was detected in normal and malignant cryptal cells (Figure 1C,D). We confirmed the membrane localization of CA IX in cryptal cells of CRC. Since we detected the β2-AR mainly in tumour regions of cryptal cells, unlike β1-AR, which was located in non-tumour regions, we assume that a more important role is played by β2-AR.

### 2.3. Beta-Blockade by Propranolol Influences Extracellular pH of Colorectal Cancer Cells in Hypoxia

**Figure 2:**
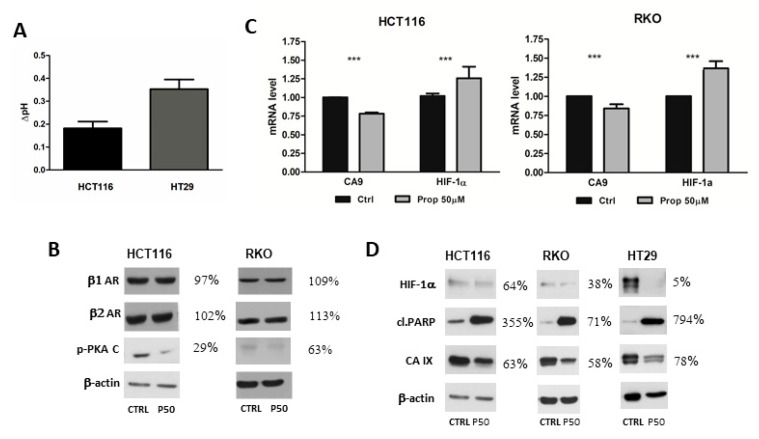
Expression of β1 and β2-AR after beta-blockade by propranolol. (**A**) Propranolol mediated alkalization of extracellular pH in hypoxia. Colorectal cancer cells HCT116 and HT29 were treated with propranolol under hypoxic conditions for 24 h. Graph shows a difference between pH of propranolol treated samples and pH of appropriate controls (mean±standard deviation, n = 3) (**B**) Western blot analysis of β1 and β2-AR levels in HCT116 and RKO cells. No significant differences in the levels of β1 and β2 receptors were detected. Beta-blockade by propranolol decreased level of phosphorylated form of catalytic subunit of PKA under hypoxic conditions. (**C**) Graph of the results of quantitative PCR (qPCR) analyses. HCT116 and RKO cells were cultured in hypoxic conditions for 24 h. The expression of *CA9* decreased after propranolol treatment. The mRNA level of HIF1α increased. The graph shows changes in mRNA levels normalized to actin. The results (mean ± stdev) represent the mean from three independent biological experiments, all performed in triplicates. Statistical significance was analyzed using the Student’s *t*-test and expressed as a *p*-value (*** *p* < 0.001) (**D**) Immunoblotting of CA IX and HIF1α proteins after treatment of colorectal cancer cell lines RKO, HCT116 and HT29 with propranolol under hypoxic conditions. Levels of CA IX and HIF1α in hypoxia decreased after beta-blockade in all three analysed cell lines and level of cleaved PARP increased. % values describe a difference in the signal of propranolol treated samples in comparison to the controls which were set as 100%. P50 represents propranolol at the concentration 50 µM.

Stimulation of β-adrenergic receptors by catecholamines activates the cAMP-protein kinase A (PKA) intracellular signalling pathway. PKA plays an important role in the regulation of CA IX activity. Active PKA phosphorylates Thr443 in the intracellular tail of CA IX and then mediates inside-out signalling to the extracellular catalytic domain that leads to the activation of the CA IX catalytic performance [38]. By measuring the pH of culture medium, we examined whether a decrease in PKA activity caused by propranolol would be accompanied by a decrease in CA IX activity. We found that in both colorectal cancer cell lines HCT116 and HT29, after propranolol treatment under hypoxic conditions for 24 h, the extracellular pH was more alkaline compared to the pH of the medium from the control cells cultivated in hypoxia with only an appropriate amount of DMSO (Figure 2A).

### 2.4. Beta-Blockade by Propranolol Decreases Level of Phosphorylated form of Catalytic Subunit of PKA under Hypoxic Conditions

Changes in the tumour microenvironment are rapidly evaluated by the cells and these respond by alterations in the expression of a wide spectrum of genes. In the treatment of CVD, the administered therapy by beta-blockers may affect all tissues that contain a target protein or target receptor, in this case β1 and β2-AR. Sensitivity to propranolol does not always correlate with the expression levels of β-adrenergic receptors [23,51]. Our results confirmed these claims and we saw no significant differences in the levels of β1 and β2 receptors detected by western blotting in HCT116 cells and RKO cells (Figure 2B). Therefore, we analysed the effect of propranolol, a non-selective beta-blocker, on downstream signalling pathway. Activation of PKA via phosphorylation plays an important role in the β-adrenergic pathway. cAMP/PKA pathway is an intracellular signalling system involved in regulation of many functions of eukaryotic cells. We investigated the effect of propranolol on the level of phosphorylated form of the catalytic subunit of PKA. The colorectal cancer cell lines HCT116 and RKO were treated with propranolol (50 µM) under hypoxic conditions (1% O_2_) for 24 h in monolayer. By western blotting and immunodetection with specific antibodies we found that propranolol reduced the amount of activated form of PKA (Figure 2B) in HCT116 and RKO cell lines under hypoxic conditions.

### 2.5. Propranolol Decreases Expression of CA IX in Hypoxic Colorectal Cancer Cells Cultivated in Monolayer

Next we tested the impact of propranolol on the expression of *CA9* and *HIF1α* genes. To quantify differences in mRNA expression qPCR was done. When normalized to *β-actin*, mRNA expression of *CA9* was lower after treatment of HCT116 and RKO cells with propranolol under hypoxic conditions (Figure 2C). Because *CA9* is one of the HIF1 target genes, we measured mRNA level of *HIF1α* subunit of HIF1 by qPCR. After normalization, we found an increased level of *HIF1α* in both analysed colorectal tumour cell lines after blockade by propranolol under hypoxia (Figure 2C). Results of qPCR were also evaluated on protein level using western blot. The colorectal cancer cells HCT116, RKO and HT29 were cultivated in a monolayer under hypoxic conditions (1% O_2_) and in the presence of propranolol (50 μM). Using immunoblotting and anti-CA IX specific antibody M75 [52], we revealed that propranolol reduced the level of CA IX under hypoxia (Figure 2D) in all three analysed cell lines. As CA IX is one of the best hypoxia-responsive targets and its transcriptional activation depends on HIF1, we determined whether a decrease of CA IX is associated with HIF-1. The results (Figure 2D) showed that propranolol (50 μM) significantly affects the level of the regulatory α subunit of the transcription factor HIF-1 under hypoxic conditions in HCT116, RKO as well as in HT29 cancer cells. Blockade of β-AR by propranolol prevents hypoxia-mediated response via HIF1.

As functional transcription factor HIF1 is important for the adaptation of tumour cells to hypoxic stress, we analysed if propranolol promotes apoptosis of tumour cells. Poly (ADP-ribose) polymerase-1 (PARP1) is a major member of the PARP superfamily that is involved in DNA damage signalling and other important cellular processes. The apoptosis marker, cleaved PARP, was analysed by western blotting and we detected a higher cleaved PARP level in response to propranolol in hypoxic conditions (Figure 2D). These data suggest that propranolol induces apoptosis in colorectal cancer cells HCT116, HT29 and RKO.

### 2.6. Propranolol Influences Adaptation to Hypoxic Microenvironment by Decreased Levels of HIF1α and CA IX in 3D Spheroids

Culturing cells in a monolayer undoubtedly has many advantages but it does not sufficiently reflect the composition of tumour. Therefore, we investigated the effects of propranolol in spheroids. Cancer cell spheroids, known as multicellular tumour spheroids, represent avascular tumour nodules. 3D spheroids are widely used for drug screening and studies of tumour growth, proliferation, transport properties and recapitulate cell–cell interactions between tumour cells and the microenvironment. Larger spheroids form gradients of oxygen and nutrients that often result in the formation of a necrotic core similar to those in poorly vascularized tumours. Spheroids also demonstrate proliferation gradients and zones reminiscent of tumours [53].

**Figure 3:**
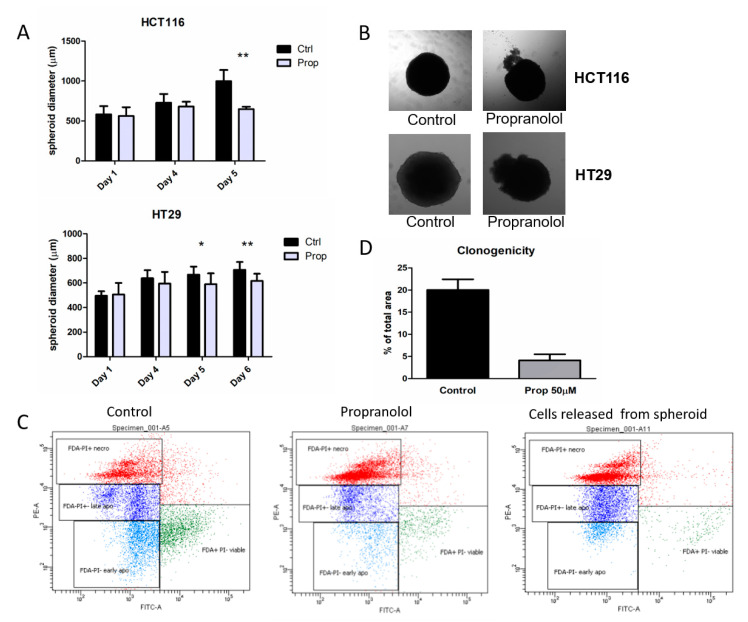
Propranolol influences adaptation to hypoxic microenvironment, reduces growth of spheroids and increases apoptosis in HCT116 and HT29 3D models. (**A**) Graph of the growth of HCT116 and HT29 spheroids in time, with/without propranolol treatment (50 µM). Spheroids were cultivated individually and regularly measured using a Zeiss Axiovert 40 CFL microscope. Propranolol slowed the growth of HCT116 as well as HT29 spheroids compared to controls. The graph gives mean±standard deviation of spheroid diameter measurements of at least 7 spheroids (* *p* < 0.05), (** *p* < 0.01). (**B**) Representative images of the morphology of HCT116 and HT29 spheroids after 5 days of treatment with beta-blocker propranolol (50 µM). After 5 days of beta-blockade the spheroids released clusters of cells from the inside. (**C**) Flow cytometry analysis of living, apoptotic and necrotic cells from HCT116 spheroids as well as of cell clusters extruded from spheroid. Propranolol treatment led to a reduction of viable cells within the spheroids, which was accompanied by an increase in apoptotic and necrotic populations. Cells extruded from propranolol treated spheroid were predominantly necrotic. (**D**) Propranolol significantly decreased the clonogenic potential of cells derived from spheroids subjected to beta-blockade compared to cells derived from control HCT116 spheroids. At the end of 5-day treatment, the spheroids were trypsinized, plated and cultured for 14 days in normoxic conditions.

Since RKO cells are unable to form and grow in spheroids, we have further analysed HCT116 and HT29 spheroids. Spheroids from HCT116 and HT29 cells were formed in hanging drops for 4 days and subsequently treated with propranolol (50 μM) for additional 5 days. Spheroids were cultivated individually and regularly measured using a Zeiss Axiovert 40 CFL microscope. Propranolol slows the growth of HCT116 as well as HT29 spheroids compared to controls cultured only with an adequate amount of DMSO (Figure 3A). After 5 days of beta-blockade, we observed that propranolol not only retarded spheroid growth but treated spheroids released clusters of cells from the inside (Figure 3B). As escape of cells from spheroids could be potentially linked to their invasive capacity, we further analysed the extruded cell clusters and found them unable to adhere and further proliferate in monolayer.

Spheroids as well as clusters of cells removed from HCT116 spheroids were analysed by flow cytometry to monitor the number of living, apoptotic and necrotic cells. Analysis of cell clusters extruded from spheroids showed that these cells are predominantly necrotic (53.9%). Only about 2% were living cells. The rest was cells in early and late apoptosis (Figure 3C). Propranolol treatment also led to a reduction of viable cells within the spheroids, which was accompanied by an increase in apoptotic and necrotic populations (Figure 3C). While the control spheroids contained only 27% of necrotic cells, propranolol-treated spheroids were formed by more than half necrotic cells (56.9%). Observed increase in the number of necrotic cells after propranolol confirms that blocking β-AR reduces the ability to compensate the changes in the spheroid microenvironment and a large percentage of cells surrender to the apoptosis and necrosis.

The effect of propranolol on the ability of a single cell to grow into a colony and proliferate was evaluated using colony forming assay. This method is used to assess the effectiveness of drugs as well as the ability of a cell to proliferate indefinitely, thereby retaining its reproductive ability to form a large colony or a clone. At the end of the experiment on the sixth day of treatment, the spheroids were trypsinized and plated. After 14 days of cultivation in monolayer under normoxia, we observed a significant decrease in the colony-forming ability of cells derived from spheroids subjected to beta-blockade compared to cells derived from control HCT116 spheroids. Results summarized in the Figure 2D clearly demonstrate that only a small fraction of cells formerly exposed to beta-blockade retains the capacity to produce colonies. These results confirm an important role of propranolol in the process of adaptation to changes in the tumour microenvironment simulated by cultivation in 3D spheroid.

**Figure 4:**
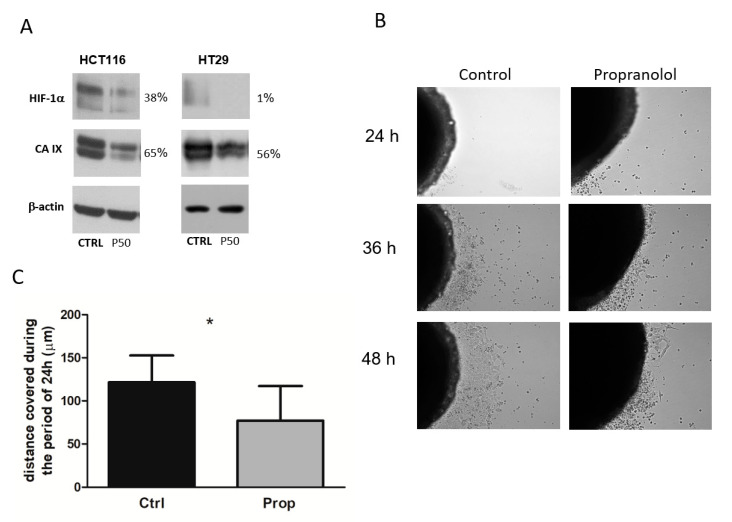
Propranolol decreases level of HIF1α and CA IX in 3D spheroids and affects migration properties of colorectal cancer cells. (**A**) Western blot analysis of CA IX and HIF1α levels. Spheroids were treated with beta-blocker propranolol for 5 days, lysed and expression of CA IX and HIF1α was analysed by immunodetection. The results demonstrate decreased level of CA IX after beta-blockade by propranolol in HCT116 and HT29 spheroids. Levels of HIF1α, the major transcriptional regulator of CA IX expression, were also reduced. % values describe a difference in the signal of propranolol treated samples in comparison to the controls set as 100%. (**B**) Representative images of time lapse microscopy analysis. In the first hours we observed a release of cells that were unable to adhere on the culture plate surface especially from spheroids after beta-blockade. Propranolol clearly decreased migration of living cells from HCT116 spheroids. (**C**) Graph displaying distance covered by cells migrating from the HCT116 spheroids with/without propranolol treatment (mean±standard deviation, * *p* < 0.05).

3D cultures are able to better control and mimic tumour microenvironment and represent a more adequate model for studying effects of drugs. As CA IX is an important component of the tumour microenvironment and the above-described results demonstrate functional consequences of propranolol, we wondered whether propranolol affected the expression of CA IX in 3D model. Spheroids were treated with beta-blocker propranolol for 5 days, lysed and expression of CA IX was analysed by western blot and immunodetection. The results (Figure 4A) clearly demonstrate decreased level of CA IX after beta-blockade by propranolol in HCT116 and HT29 spheroids. Results of immunodetection also showed reduction in the levels of HIF1α, the major transcriptional regulator of CA IX expression (Figure 4A). These findings suggest that propranolol significantly disrupts the processes of tumour cell adaptation to microenvironmental stress. Especially under insufficient oxygen concentration, the reduced level of HIF1α and, consequently, decreased level of functional transcription factor HIF1 lead directly to a disruption of hypoxia-regulated signalling.

### 2.7. Propranolol Decreases Migration Properties of Colorectal Cancer Cells

Previous results showed the effect of propranolol on morphology, growth and adaptation to microenvironmental changes within the spheroid. Cell migration is a key hallmark of malignant cells that contributes to the progression of cancers from a primary, localized mass to an invasive and/or metastatic phenotype. Therefore, we studied the functional consequences of propranolol on cell growth in 3D systems. Considering cell motility as an essential feature of both invasion and dissemination, we studied a possible effect of propranolol on migration of cells from spheroids. During time lapse microscopy analysis we found out that propranolol significantly reduced the ability of cells to migrate from the spheroid to its surroundings (Figure 4B,C). Moreover, in the first hours we observed a release of cells that were unable to adhere on the culture plate surface especially from spheroids after beta-blockade. Propranolol clearly decreased migration of living cells from HCT116 spheroids and these results suggest that propranolol may reduce the migration ability of cells from the tumour.

### 2.8. Distribution of CA IX in 3D Spheroids Is Polarized

**Figure 5:**
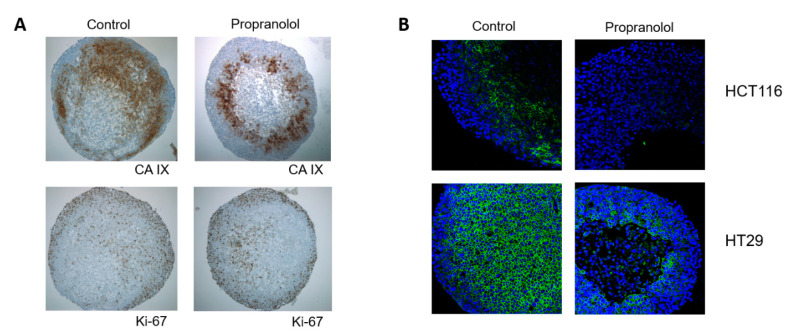
Propranolol influences adaptation of cells to hypoxic microenvironment and decreases level of CA IX. (**A**) Immunohistochemical analysis of CA IX and Ki-67 expression and localization in HCT116 spheroids sections. CA IX was expressed in the hypoxic centre and perinecrotic regions, a strong expression was observed in the regions very close to the spheroid surface. Expression of CA IX was detected in areas of proliferative Ki-67 positive cells. Localization and distribution of CA IX in spheroids is, independently of propranolol treatment, polarized and non-uniform. Proliferative (Ki-67 positive) and CA IX positive cells are localized in the field opposite to necrotic cells area. (**B**) Immunofluorescent analysis of CA IX expression in HCT116 and HT29 spheroids. Reduced expression of CA IX was confirmed also by immunofluorescence. The central regions of the HCT116 and HT29 spheroid were necrotic after 5 days of β-AR inhibition, while the integrity of the control spheroids remained intact.

Multicellular models more faithfully simulate conditions in tumour tissue in terms of creating gradients of oxygen, pH, nutritional, and waste products. CA IX is found in tissues mainly in hypoxic areas, areas after hypoxic stress and in perinecrotic regions. In connection with the above presented results, we analysed whether and how propranolol affects distribution of CA IX in a spheroid. Spheroids, propranolol treated and controls, were fixed in Carnoy’s fixative and embedded in paraffin. Then 4 µm spheroid sections were analysed using immunohistochemistry. Our results showed that CA IX was expressed not only in the hypoxic centre or in perinecrotic regions but a strong expression was also observed in the regions very close to the spheroid surface. Expression of CA IX was detected in areas of proliferative Ki-67 positive cells (Figure 5A). Localization and distribution of CA IX in spheroids is, independently of propranolol treatment, polarized and non-uniform. Proliferative (Ki-67 positive) and CA IX positive cells are localized in the field opposite to necrotic cells area. Necrotic cells are extruded from central hypoxic regions out of a spheroid. This may represent a way of adaptation to changes in the tumour microenvironment where the proliferative Ki-67 positive cells are in the frontal invasive region and dead cells unable to manage stressful conditions are eliminated from spheroids. CA IX seems to confer the adaptation advantage for the acidic and hypoxic environment. If the hypoxic signalling pathway is inhibited, the cell removal process is accelerated. Reduced expression of CA IX in sections of HCT116 as well as HT29 spheroids was also confirmed by immunofluorescence. Our results showed a strong expression of CA IX in HT29 spheroids and reduction of CA IX after beta-blockade (Figure 5B). The central regions of the HCT116 and HT29 spheroids were necrotic after 5 days of β-AR inhibition, while the integrity of the control spheroids remained intact.

### 2.9. Propranolol Affects Cell Stress Proteome in Spheroids

**Figure 6:**
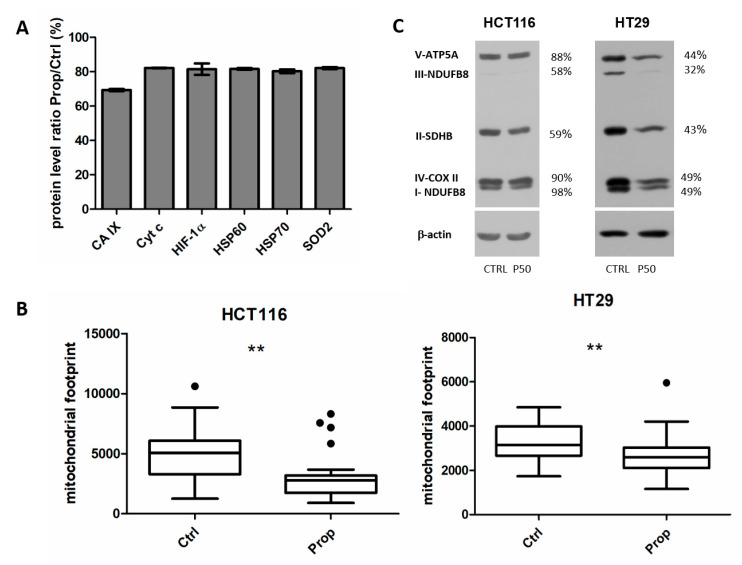
Propranolol reduces number of mitochondria and mitochondrial ox-phos enzymes. (**A**) Cell stress related proteins differentially expressed in spheroids after beta-blockade by propranolol. Using the Proteome Profiler Human Cell Stress Array Kit we analysed protein lysates from 15 spheroids from HCT116 cells treated for 5 days with propranolol and appropriate controls. The screening analysis showed 6 differentially expressed proteins. The results confirmed decreased levels of HIF1α and CA IX and of other proteins involved in mitochondrial metabolism and in oxidative phosphorylation. (**B**) Analysis of the amount of mitochondria using a Mito tracker MitoRed. Confocal analysis colorectal cancer cells monolayers cultured in hypoxia showed a decreased mitochondrial footprint, that is, the area of cell consumed by mitochondria signal, in samples treated by propranolol in both HCT116 and HT29 cells. Images were processed by MiNA toolset in ImageJ 1.41g. Box plot graphs of the measurements of at least 40 control and treated cells are displayed (** *p* < 0.01). (**C**) Western blot analysis of oxidative phosphorylation (OXPHOS) proteins in spheroid samples from HCT116 and HT29 cells. After propranolol treatment, the levels of proteins essential for the process of oxidative phosphorylation were reduced. % values describe a difference in the signal of propranolol treated samples in comparison to the controls set as 100%.

Beta-blockers are generally used to inhibit the effects of the stress hormones catecholamines. Our results pointed out that after beta-blockade with propranolol, spheroid cells are not able to respond adequately to changes in the oxygen gradient produced during cell growth in the 3D structure. To evaluate the spectrum of proteins affected by beta-blocker propranolol in spheroids, we performed Cell Stress proteome profiling array (PPA). The Proteome Profiler Human Cell Stress Array Kit is a membrane-based sandwich immunoassay that detects 26 human cell stress-related proteins simultaneously. We analysed protein lysates of 15 spheroids from HCT116 cells treated for 5 days with propranolol and appropriate controls. The screening analysis showed (Figure 6A) differential expression of 6 proteins. The results confirmed our previous results of β-AR blockade inhibitory effects on HIF1α and CA IX. Proteins with distinctly altered expression were involved predominantly in mitochondrial metabolism-superoxide dismutase (SOD2), Hsp60, Hsp70 and in oxidative phosphorylation–cytochrome c.

### 2.10. Propranolol Reduces Number of Mitochondria and Mitochondrial ox-phos Enzymes

Since PPA results confirmed the involvement of mitochondria in the response to β-AR inhibition, our further efforts led to the analysis of the amount of mitochondria using a Mito tracker MitoRed. MitoRed is a cell membrane permeable Rhodamine-based dye which localizes to mitochondria and emits red fluorescence. The interaction of MitoRed with mitochondria depends on the membrane potential of the mitochondria. Confocal analysis of propranolol treated/control colorectal cancer cells HCT116 and HT29 cultured in hypoxia and stained by MitoRed showed a decreased mitochondrial footprint, that is, the area of cell consumed by mitochondria signal, in samples after inhibition of β-AR by propranolol (Figure 6B).

Due to the altered amount of mitochondria, we went on to analyse the levels of oxidative phosphorylation enzymes by western blotting using a cocktail of specific antibodies. We found that the levels of proteins essential for the process of oxidative phosphorylation were reduced in spheroid samples from HCT116 and HT29 spheroids after propranolol treatment (Figure 6C).

Our results have shown that propranolol significantly affects the amount of mitochondria as well as the amount of enzymes involved in oxidative phosphorylation.

## 3. Discussion

Comorbidity or the coexistence of various chronic illnesses, has an important impact on the management and prognosis of cancer patients. The results of the De Marco et al. (2000) study have clearly demonstrated a high prevalence of severe comorbidities in colorectal cancer patients [54]. From the group of unselected colorectal cancer patients, approximately 35% of patients below the age of 70 and 61% of patients over 70 years of age had a serious comorbidity. Moreover, chemotherapy significantly increases the risk of CVD and cardiotoxicity during oncological treatment of cancer patients [8].

Several studies have analysed a possible relationship between stress, catecholamine levels, beta-blocker use and cancer incidence or outcome. Unfortunately, with partly controversial results [27,32,55]. The reasons can be multiple, for example the type of stress (acute, chronic or exercise-induced stress) connected with different catecholamine compositions, the type of tumour, phases during tumorigenesis, stages, anti-cancer therapy, as well as type of administered beta-blocker [32,56]. The molecular and cellular mechanisms by which stress increases the risk of certain types of cancer and their prognosis remain understudied. Above mentioned reasons are undoubtedly important but it is equally important to know whether the non-oncologic concomitant medication by beta-blockers affects the behaviour of the tumour itself and how it influences the tumour microenvironment. Therefore, our work was focused on elucidating the effect of a non-selective beta-blocker propranolol on colorectal cancer cells cultured in a 3D multicellular spheroid model. The spheroid culture system is a model used increasingly in studies of function, metabolism and in toxicological assessment of drugs [57,58]. Naturally arising gradients of oxygen, pH, nutritional and waste products as well as cell-cell interactions in a 3D model more faithfully simulate the tumour microenvironment. Our results of β-AR expression in patients’ tissue samples confirmed that β1 and β2 AR are expressed in colorectal tumour tissues. While the expression of β1-AR was associated predominantly with mastocytes and smooth muscle of vessels in non-malignant regions, expression of β2-AR was detected in normal and malignant cryptal cells. We confirmed a strong membrane localization of CA IX in cryptal cells of malignant colorectal cancer samples and a week expression in cryptal cells of healthy tissues. The presence of β2-AR in tumour-altered regions of the colon suggests a possible effect of non-oncological treatment with propranolol on tumour tissues. When canonically activated, the β-AR induce intracellular signal transduction pathways through triggering cyclic adenosine monophosphate and cAMP-dependent protein kinase A cascades. Our previous study has shown that the catalytic activity of CA IX is dependent on threonine phosphorylation (Thr443) in the intracellular domain of CA IX by PKA in hypoxia [38]. Hypoxia induces the cAMP signalling pathway at several upstream and downstream levels. HIF1 seems to play an important role and to contribute also to an increased expression of isoforms ADCY VI and ADCY VII, resulting in higher cAMP generation that affects PKA function, with impact on cell migration and pH regulation [59]. CA IX is a tumour-associated, hypoxia-regulated protein whose expression has been manifested in a broad spectrum of solid tumours [36]. As a pH regulating enzyme, CA IX plays a key role in maintaining an acidic extracellular pH under hypoxia. Competitive inhibition of β-adrenergic receptors blocks activation of intracellular signalling pathways. Our results clearly demonstrate the impact of beta-blockade by propranolol on the extracellular pH of colorectal cancer cells monolayers under hypoxic conditions. An increase in the extracellular pH as a result of antagonistic effect of propranolol can occur due to reduced PKA activity and consequent decrease in CA IX activity. Manipulation of the extracellular and/or intracellular pH of tumours may have extensive potential in the therapy of cancer. Numerous studies have explained that the aerobic glycolysis of cancer cells is commonly linked to chronic overactivation of the hypoxia-inducible factor-1 (HIF1), which boosts the expression of glycolytic enzymes and pyruvate dehydrogenase kinase-1 (which inhibits pyruvate dehydrogenase and thus accelerates a conversion of pyruvate to lactate) and promotes mitochondrial autophagy [60,61,62].

Our results have shown that propranolol not only increases extracellular pH and thus disrupts the tumour pH homeostasis but beta-blockade by propranolol also decreases CA IX level. Thus, CA IX may be affected by propranolol both at the level of activity through a decrease in PKA activity but also at the level of expression. CA IX is one of the best responders to hypoxia and is transcriptionally regulated by hypoxia through HIF1 binding to an HRE sequence localized immediately in front of the transcription initiation site [37].

CA IX directly cooperates in many acidosis-induced features of tumour phenotype as demonstrated by manipulating its expression. CA IX can function as a pro-survival factor protecting tumour cells from hypoxia and acidosis, as a pro-migratory factor facilitating cell migration and invasion, as a signalling molecule transducing extracellular signals to intracellular pathways and converting intracellular signals to extracellular effects on adhesion, proteolysis and other processes [36]. Therefore, changes in CA IX levels can directly affect multiple processes resulting in changes in the tumour microenvironment. Since hypoxia is the main driving force behind CA IX expression, we analysed the impact of propranolol treatment on the transcriptional factor HIF1. As we detected a decrease in CA IX levels, we wondered whether this result was directly related to changes in the accumulation of the regulatory subunit HIF1α. Our findings that not only CA IX but also HIF1α level is decreased by propranolol led us to suggest that beta-blockade directly affects the ability of tumour cells to adapt to hypoxic stress. The participation of β-AR signalling in the regulation of HIF1 has been indicated by several authors. Wu et al., 2016 described that β2-AR signalling negatively regulates autophagy in an Akt-dependent manner, leading to HIF1α stabilization [16]. On the other hand, the inhibition of β2-AR signalling by β2 selective antagonist ICI118551 or knockdown of β2-AR expression, led to enhanced autophagy, HIF1α destabilization and tumour growth suppression. In the study of hemangioblastoma cells from patients bearing mutation in von Hippel-Lindau gene, authors described downregulation of HIF-dependent transcription by propranolol under hypoxic conditions and its impact on proliferation [63].

Publications describing the effect of propranolol on the hypoxic pathway were performed in simulated hypoxia and at monolayer. In our experimental model, there is a spontaneous formation of an oxygen gradient, which more accurately mimics the tumour situation and tumour microenvironment. In larger spheroids, the absence of oxygen results in the formation of a necrotic core similarly as in poorly vascularized tumours. We observed that the morphology of the spheroids differed considerably during the treatment with propranolol. The beta-blockade by propranolol reduced the proliferation of cells in the spheroid and slowed their growth. In our work, we also pointed out the pro-apoptotic effect of beta-blockade by propranolol. The spheroids cultured in the presence of propranolol for 5 days were unable to manage the absence/decrease of the HIF-mediated adaptation pathways and we observed release of clusters of apoptotic or necrotic cells from the spheroids. The integrity of the treated spheroids was impaired. Results of flow cytometry confirmed the effects of beta-blockade on apoptosis activation. An analysis of cells derived from spheroids revealed an increase in the number of cells in apoptosis as well as a significant number of necrotic cells after exposure to propranolol. The impact of propranolol on the survival and proliferation of colorectal tumour cells was also confirmed by a significantly reduced ability of single cells to grow into colonies. Through this assay, we assessed the capability of clonogenic survival. The anti-proliferative effect of beta-blockers has been suggested in several studies. Coelho et al. (2015) explored the impact of a number of β-AR agonists and antagonists on the proliferation of HT29 colon adenocarcinoma cells [64]. Chin et al. (2016) also investigated the effect of beta-adrenergic receptor antagonists, including propranolol, in a panel of colorectal cancer cells lines and they showed inhibition of the viability of colorectal cancer cells [65]. Moreover, propranolol induced G1-phase arrest in these cells. Decreased viability, proliferation and activation of apoptosis by propranolol has also been reported in tumours of the head and neck [66], pancreas [67], prostate [68] and breast [69].

Our findings of the distribution and localization of CA IX protein within spheroids were very interesting. We found that the distribution of CA IX protein was not uniform across a spheroid. In a spheroid, not only a gradient of oxygen and pH occurs but also a gradient of proliferation. Spheroids displayed a layered cell distribution analogous to that observed in solid tumours. Well-nourished cells in the outer layer exhibit high proliferation whereas the middle layer is characterized by quiescent cells and the depleted core may contain necrotic cells and acellular regions. However, our findings have shown that the layered segmentation is not strict. As the spheroid grows and a necrotic core is formed, the layered division becomes polarized. We observed that necrotic cells from the central region of the spheroid are extruded and removed on one side of the spheroid. Quiescent and proliferating cells are in the opposite field. Such distribution of cells and regions within the spheroid was observed in both control and propranolol treated spheroids. In control spheroids, we observed these effects with time. This seems to be a way of clearing the central spheroid regions from necrotic cells, since the spheroids were made up of a single cell type and did not include immune cells or stromal components. Overexpression of CA IX was detected mainly in hypoxic and perinecrotic areas of spheroids and it overlapped with the expression of the proliferation marker Ki-67.

Determining migratory phenotype of tumour cells and understanding molecular mechanisms is fundamental for novel strategies in diagnosis, prognosis, drug development and treatment of cancer. Metastasis is the main cause of cancer lethality, 90% of deaths from solid tumours can be ascribed to metastatic dissemination [70]. In time-lapse experiments we observed a significantly slower migration of cells from spheroid to the environment. Several studies have highlighted the stimulatory effects of catecholamines on migration of prostate cancer cells [71,72]. On the contrary, Huang et al., (2019) presented no direct effect of a non-selective β-AR agonist on migration and invasion of human prostate cancer PC-3 and DU145 cells [73]. The recent study of in vivo metastatic model confirmed the activation of the β2-AR signalling pathway during chronic stress and promotion of liver and lung metastasis in nude mice [74]. A proper control of pH is critical also for cell migration and invasion. CA IX is localized in protruding fronts of migrating cells together with other constituents of pH regulating machinery and it provides a benefit to tumour cells in form of survival advantage and invasive behaviour, particularly under low concentration of oxygen and acidosis. CA IX deficiency is associated with reduced migration, while its overexpression has opposite effects [75]. Our results directly support the significant inhibitory effect of propranolol on the ability of colorectal tumour cells to migrate from spheroid. β-AR signalling plays an important role in the response to stress. Our Cell Stress Proteome profiling also confirmed previously mentioned downregulation of CA IX and HIF1α proteins by propranolol. The results further showed the involvement of mitochondria in the cell response to blockade of β-AR by propranolol. Mitochondria are essential organelles for many fundamental cellular processes, such as energy production, fatty acid β-oxidation, metabolite synthesis, iron and calcium homeostasis and programmed cell death. Accumulating evidence suggests that the development of cancer requires an abundance of ATP for unlimited proliferation and/or survival. This means that mitochondrial metabolism is necessary for tumorigenesis. Here we showed a direct effect of propranolol on the amount of mitochondria in colorectal tumour cells under hypoxia. The role of mitochondrial metabolism in response to beta-blockade seems to be important. According to Lucido et al. the antitumor activity of propranolol is associated with inhibition of mitochondrial metabolism in some tumour types [49]. Seahorse analysis showed that propranolol caused a significant reduction in the baseline oxygen consumption rate (OCR) and the OCR associated with ATP production, as well as a reduction in the maximum OCR in the lung metastasis derived from primary head and neck cancer with hyperactive mitochondrial metabolism. Proliferation and migration are energy demanding processes and attenuation of mitochondrial respiration could explain the effect of beta receptor antagonists. Recent studies using a combination of propranolol with glycolysis inhibitors have shown a synergistic effect of glucose deprivation and propranolol on its antitumor activity. The use of propranolol together with a clinically available glycolysis inhibitor dichloroacetate resulted in a dramatic decrease in tumour cell metabolism, inhibition of proliferation and induction of apoptosis in head and neck cancer lines [50]. Treatment of prostate tumour cells with a combination of propranolol and 2-deoxy-d-glucose resulted in a massive accumulation of autophagosomes. This combination also effectively prevented tumour cell proliferation, induced their apoptosis, altered mitochondria morphology and inhibited their activity, increased ER stress in vitro and also suppressed tumour growth in vivo [48].

The above-described effects of beta-blockade by propranolol on colorectal tumour cells in terms of reduced adaptation to hypoxic stress, reduced proliferation, viability as well as limited ability to migrate indicate the need to consider the impact of administered beta-blocker therapy when choosing appropriate anti-tumour therapy.

## 4. Materials and Methods

### 4.1. Cell Culture and Growth Conditions

Colorectal cancer cells HCT116, HT29, RKO were routinely cultured in DMEM culture medium supplemented with 10% FCS (HyClone Laboratories) and gentamicin (Sandoz). Experiments in hypoxia (1% O_2_) were done in a humidified anaerobic workstation (Ruskinn Technologies) with 5% CO_2_, 10% H_2_ and 84% N_2_ at 37 °C. All cell lines were purchased from ATCC and regularly tested for Mycoplasma contamination. Cells were treated with beta-blocker propranolol dissolved in DMSO in the final concentration 50 µM. Control cells were cultivated in the presence of an appropriate volume of DMSO. At the end of each experiment, pH of the culture medium was immediately measured by pH meter Seven2Go, S2, Mettler Toledo and microelectrode InLab Nano (Switzerland). The pH values were subsequently normalized to total protein concentration of each sample.

### 4.2. Western Blotting

After treatment with propranolol cells grown in monolayers or spheroids were rinsed with PBS, resuspended in ice-cold lysis buffer (50 mM TrisHCl pH 7.4; 150 mM NaCl; 1% Triton X100; 0.05% NaDOC; 1 mM EDTA; 0.1% SDS) supplemented with protease (Roche) and phosphatase inhibitors cocktail (Sigma Aldrich, St. Louis, MO, USA) and incubated on ice for 15 min. Spheroids were disrupted by sonication. Protein concentrations were quantified using the BCA protein assay reagents (Pierce). The protein extracts (50 µg/lane) were resolved in 10% SDS-PAGE and transferred to a PVDF membrane (Immobilon TM-P, Millipore). After blocking with 5% non-fat dry milk in 0.1% Tween in PBS, membranes were incubated with primary antibodies: M75 hybridoma medium [52] (for CA IX detection, 1:3 in blocking buffer, 1 h, RT); anti-β-actin (Cell Signalling, Danvers, MA, USA, 1:5000 in 3% BSA in TBS-T buffer, 1 h, RT); anti-HIF1α (BD Transduction Laboratories, San Jose, CA, USA, 1:250 in 3% BSA in TBS-T buffer, O/N, 4 °C); anti-cleaved PARP (Cell Signalling, 1:1000, 1 h RT), anti-phospho-PKA (Cell Signalling, 1:1000 in 3% BSA in TBS-T buffer, 1 h RT), total OXPHOS Antibody Cocktail (Abcam, Cambridge, UK, 1:500 in 3% BSA in TBS-T buffer, 1 h, RT), anti-β1 adrenoreceptor (Thermo Fisher Scientific, Waltham, MA, USA, 1:500 in 3% BSA in TBS-T buffer, 1 h, RT), anti-β2 adrenoreceptor (Abcam, 1:1000 in 3% BSA in TBS-T buffer, 1 h, RT). After washing, membranes were incubated with peroxidase-conjugated anti-mouse or anti-rabbit antibody (Dako, Glostrup, Denmark, 1:5000 in blocking buffer, 1 h, RT). Protein signals were visualized using an enhanced chemiluminescence kit (GE Healthcare Bio-Sciences, Piscataway, NJ, USA). All protein bands were quantified in ImageJ software (using Analyse-Gels tool) and all results were normalized to actin, propranolol treated bands are compared to signals of appropriate control bands which are set as 100%. Western blot analyses were repeated three times.

### 4.3. Proteome Profiler Array

Proteome profiler array analyses (PPA) analyses were performed using the Human Cell Stress ArrayKit (R&D Systems, Minneapolis, MN, USA) according to the manufacturer’s recommendations. Briefly, spheroids were formed in hanging drops for 4 days and subsequently treated with propranolol for 5 days. 15 spheroids from each condition were lysed and total proteins were allowed to interact with membranes with spotted antibody arrays overnight at 4 °C. Proteins were quantified by measuring the accumulated pixel density of the individual spots and adjusted based on reference spots using the ImageJ software.

### 4.4. Flow Cytometry Analysis of Cell Viability

The propranolol treated and control spheroids were washed with PBS. Spheroids were dissociated into single cell suspension after incubation in TrypLE Express for 15 min at 37 °C. Cells released from spheroids were harvested and washed twice in PBS. Determination of apoptotic cell count was done according to the number of cells with their cell membrane impermeable to propidium iodide (PI) and low Fluorescein diacetate (FDA) fluorescence, necrotic cells were determined as those with cell membrane permeable for PI. Living cells were labelled with FDA.

Approximately 5 × 10^5^ cells were resuspended in 400 μL of PBS/0.2% BSA containing 10 nM of FDA (5 mM stock in DMSO) for 20 min at room temperature. Then cells were cooled and 4 μL of PI (1 mg/mL) was added. Finally, cells were measured using Canto II (Becton Dickinson) flow cytometer and analysed by FCS Express 4.0 (De Novo Software, Glendale, CA, USA). Flow cytometry analysis was repeated three times.

### 4.5. Quantitative Real-Time PCR

Total RNA was extracted using TRI-reagent (Sigma). Reverse transcription of RNA was performed with High-capacity cDNA Reverse Transcription kit (Applied Biosystems, Foster City, CA, USA) according to the manufacturer’s recommendations. Q-PCR was performed using Maxima SYBR Green PCR Master mix (Thermo Scientific) and ran as follows: 10 min at 95 °C for initial denaturation, 40 cycles of 95 °C for 15 s and 60 °C for 1 min. Sample Ct values were normalised to β-actin. Relative expression was calculated using the ΔΔCt method. Oligonucleotides used for qPCR were as follows: *CA9 F*: 5′ TAT CTG CAC TCC TGC CCT CTG 3′, *CA9 R*: 5′ CAC AGG GTG TCA GAG AGG GTG 3′, *HIF-1α F*: 5′ GCT TGG TGC TGA TTT GTG AAC C 3′, *HIF-1α R*: 5′ GCA TCC TGT ACT GTC CTG TGG TG 3′. Amplifications were performed in triplicate and results were calculated from three independent experiments.

### 4.6. Formation of Spheroids in Hanging Drops

The spheroids were formed in 20 µL hanging drops containing 100 cells/µL in DMEM for 4 days of incubation in a humidified atmosphere on the lid of Φ100 mm Petri dish. PBS was added into the dish to prevent drying of the drops. Resulting cell aggregates/spheroids were carefully transferred to plates with a non-adherent surface and cultivated in suspension with propranolol at a concentration of 50 µM for 5 more days. Control spheroids were incubated with an appropriate volume of dimethyl sulphoxide (DMSO). The spheroids were examined with a Zeiss Axiovert 40 CFL microscope, A-Plan 5×/0.12 objective (Zeiss) and processed by Axiovision 4.8 software.

### 4.7. Clonogenic Assay

After 5 day treatment with propranolol (or without treatment) the spheroids were trypsinized, counted and dispensed into 6 well plates. Subsequently, the cells were cultured for 14 days under normoxic conditions in drug-free Dulbecco’s Modified Eagle Medium (DMEM). The colonies were then stained and fixed with methylene blue in ethanol. Results were expressed as the area fraction covered by colonies, related to the total area of a culture dish. Colonies area was quantified using ImageJ software.

### 4.8. Immunofluorescence of Spheroids

The spheroids, after treatment with propranolol as well as the corresponding controls, were washed with PBS and fixed with ice-cold methanol for 10 min at −20 °C. After washing with PBS, the spheroids were incubated with primary antibody M75 (undiluted hybridoma medium for CA IX) for 2 h at RT. The nuclei of spheroids were stained with DAPI (300 nM). After washing with PBS 2 × 30 min, the spheroids were incubated with a secondary anti-mouse Alexa 647 antibody, diluted 1: 1000 for 1 h at RT. After washing with PBS 2 × 30 min, the spheroids were analysed using confocal microscopy CQ1 (Accela), 10× objective. The images were processed using the ImageJ software.

### 4.9. Immunohistochemistry

The spheroids were fixed in Carnoy fixative (Ethanol:Chloroform:Glacial acetic acid 6:3:1) and embedded in paraffin. Patient tumour tissues were fixed in neutralized formalin and embedded in paraffin. Antigen retrieval was performed at low pH retrieval buffer (EnVision^®^ Flex Target Retrieval Solution Low pH) at PT-Link (Dako). Immunohistochemical analysis was performed on an automated immunostainer (Dako Autostainer) using DakoCytomation EnVision^®^+ System-HRP (DAB) according to the manufacturer’s instructions. For CA IX staining, the sections were labelled with M75 antibody (hybridoma medium) diluted 1:100 for 1 h at room temperature. For β1 and β2 adrenoreceptors and Ki-67 the sections were incubated with anti β1 (Invitrogen, Carlsbad, CA, USA, 1:500), anti β2 (Abcam, 1:250) overnight at 4 °C, for Ki-67 (DAKO, 1:100) for 1h at room temperature and, after washing, incubated with secondary anti mouse-HRP or anti rabbit-HRP antibody for 30 min at room temperature. Staining was visualized using 3,3’-diaminobenzidine (DAB) as a chromogenic substrate for 1 min. The sections were counterstained with Mayer’s haematoxylin and mounted in Aquamount (Merck, Darmstadt, Germany). The stained sections were examined using a Leica DM4500B microscope and images were captured by a Leica DFC480 camera.

### 4.10. In Silico Analysis

In silico analysis of β1 and β2 adrenoreceptors expression in colorectal tumour tissues was performed using Genevestigator (https://genevestigator.com) [76]. Genevestigator database integrates thousands of manually curated, well described public microarray and RNA-Seq experiments and visualizes gene expression across different biological contexts (tissues, diseases).

### 4.11. Time-Lapse Microscopy

HCT116 spheroids were incubated with propranolol (50 µM) for 5 days. On the sixth day, the spheroids were moved to a 12-well plate for the cultivation of adherent cells. Time-lapse acquisition was performed with a Zeiss Cell Observer System with controlled temperature, humidity and an active gas mixer, at 10× objective. Spheroids were in the incubation chamber at 37 °C in 21% O_2_, 5% CO_2_ atmosphere. Imaging was managed by Axiovision 4.8 software, using the Multidimensional Acquisition settings. The experiment started 60 min after spheroids began to attach (designated as time 0 min) and images were taken in the transmitted light every 60 min at least 6 different positions for each sample for 72 h. The migration of cells from a spheroid was measured as the distance that the cells reached from the edge of the spheroid. Cell migration analysis was performed from time-lapse images in the ImageJ program. The experiment was repeated three times.

### 4.12. Fluorescent Labelling and Analysis of Mitochondrial Network

HC116 and HT29 cells were seeded on round glass cover slips and cultivated in the presence of propranolol (50 µM) or with appropriate amount of DMSO for 24 h in hypoxic conditions (1% O_2_). The live cells were stained with Mitotracker (stock solution 1 mM) diluted 1:1000 for 30 min at 37 °C. Afterwards, cells were washed with PBS and fixed with 4% paraformaldehyde for 20 min at room temperature. After washing, cover slips were mounted on glass slides with mounting medium. Samples were analysed by Zeiss LSM 510 Meta confocal microscope, Plan Neofluar 40×/1.3 oil objective in the multitrack scanning mode. The images were analysed by Mitochondrial Network Analysis toolset (MiNA) in the ImageJ program [77]. At least 40 cells were analysed for each sample.

### 4.13. Ethic Approval and Patients Tissue Samples

The study protocol was approved by the Ethics Committee of Biomedical Research Centre, Slovak Academy of Sciences, Ethics approval EK/BmV-02/2016 and was in accordance with the principles of the Declaration of Helsinki. All subjects gave their written informed consent before participation. Patients’ samples were received from the University Hospital Bratislava, Ružinov Hospital.

### 4.14. Statistical Analysis

Where applicable results were analysed by two-tailed unpaired *t*-test (Student’s *t*-test) and *p* < 0.05 was considered significant. *p* < 0.05 is denoted as *, *p* < 0.01 is denoted as **.

## 5. Conclusions

Our data provide evidence and basis for a better understanding of the effects of non-oncological medication by non-selective beta-blocker propranolol on tumour microenvironment and on the adaptation of colorectal cancer cells to hypoxia. The inhibition of β-AR by propranolol enhances apoptosis and reduces metastatic potential, viability and proliferation of colorectal cancer cells cultivated in multicellular spheroids. Generally, the presence of CA IX in cancer is accompanied by the ability to survive hostile conditions, acquire metastatic inclination and gain resistance to therapy. For the first time, we demonstrated the effect of propranolol on CA IX and HIF-1α levels in a 3D multicellular model simulating a more faithful tumour mass and relations in the tumour. Propranolol decreases HIF-1α accumulation as well as CA IX levels, thereby disadvantaging colorectal tumour cells in adaptation to hypoxia and acidosis. Decision-making about targeted anti-cancer therapy should also take into account the presence and treatment of concomitant diseases. The consequences of beta-blockade by propranolol results in the impaired ability of cells to adapt to hypoxic stress by activating appropriate signalling pathways, which leads to the induction of apoptotic pathways, decreased proliferation and viability.

## Abbreviations

CA IX	Carbonic anhydrase IX
β-AR	Beta adrenergic receptor
HIF1	Hypoxia inducible factor 1
PKA	Protein kinase A
